# Rational strain improvement for surfactin production: enhancing the yield and generating novel structures

**DOI:** 10.1186/s12934-019-1089-x

**Published:** 2019-02-28

**Authors:** Fangxiang Hu, Yuyue Liu, Shuang Li

**Affiliations:** College of Biotechnology and Pharmaceutical Engineering, Nanjing Tech University, No. 30 Puzhu South Road, Nanjing, Jiangsu China

**Keywords:** Surfactin, Branched chain fatty acids, Biosynthesis, Structure, NRPS

## Abstract

Surfactin, one of the most powerful microbial surfactants, is a lipopeptide-type biosurfactant which combines interesting physicochemical properties and biological activities. However, the high cost caused by its low productivity largely limits the commercial application of surfactin. Hence, many engineered bacterium have also been used to enhance surfactin biosynthesis. This review briefly summarizes the mechanism of surfactin biosynthesis, highlighting the synthesis pathway of N-terminally attached fatty acids, and outlines the main genetic engineering strategies for improving the yield and generating novel structures of surfactin, including promoter engineering, enhancing efflux systems, modifying the transcriptional regulatory genes of surfactin synthase (*srfA)*, genomics and transcriptomics analysis, non ribosomal peptide synthetase (NRPS) domain and combinatorial biosynthesis. Finally, we discuss the future prospects of the research on surfactin.

## Background

Since green chemicals and industrial processes have become a concern for the whole society, environmentally-friendly, biodegradable biosurfactants with low toxicity have aroused great interest [1, 2]. Biosurfactants are classified into glycolipids, phospholipids, fatty acid lipopeptides, lipoproteins, polymeric surfactants and particulate surfactants based on their natural chemical structure and microbial origin [3, 4]. Lipopeptides constitute a class of antimicrobials composed of a hydrophilic peptide ring and hydrophobic fatty acid moieties [5, 6]. According to their structural characteristics, lipopeptides can be divided into cyclic lipopeptides (CLPs) and linear lipopeptides [7, 8]. The cyclic lipopeptides that have been discovered so far, including fengycin, iturin and surfactin, are mainly produced by *Bacillus subtilis* [9, 10]. These lipopeptides all have a peptide ring of 7 or 10 amino acids with a long hydrophobic fatty acid chain. The fatty acid chain lengths differ, whereby that of surfactin is C_13_–C_16_, iturin’s chain is C_14_–C_17_, and that of fengycin is C_14_–C_18_.

**Fig. 1:**
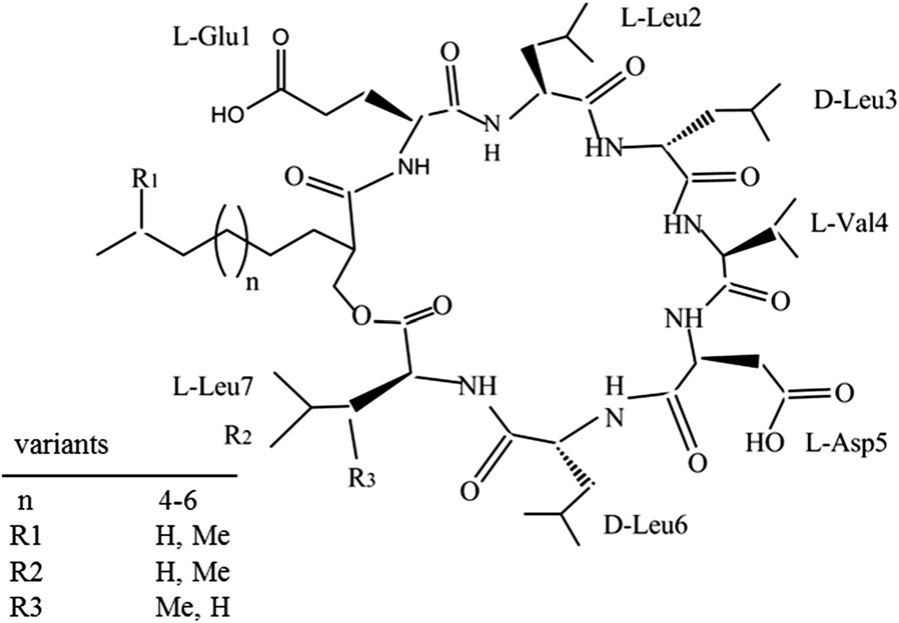
Chemical structure of surfactins [32]

Surfactin, a secondary metabolite first discovered in the culture broth of *B. subtilis* in 1968 [11], is the most well-known lipopeptide with broad-spectrum antibacterial activity. Surfactin has a ring-shaped peptide chain containing seven amino acids, and a β-hydroxy fatty acid chain of 13–16 carbon atoms as shown in Fig. 1 [12]. Because of some differences in the length of the fatty acid chain and the types of amino acids, surfactin has many congeners or isoforms [13]. Due to its unique structure, surfactin can not only lower the surface tension of water from 72 to 27 mN/m, but is also highly thermally stable and salt tolerant [14, 15]. Thus, it has great potential in both enhanced oil recovery (EOR) and the microbial enhancement of oil recovery (MEOR) [16, 17]. In addition, surfactin has been considered as a good candidate for use in bioremediation of contaminated soils and subsurface environments [18].

Although surfactin has potential therapeutic applications of anticancer therapy and environmental applications [19], it cannot compete with synthetic chemical surfactants due to its high cost and low yields. In order to reduce the cost, several waste materials such as feather hydrolysate waste, glutamate mill waste and distillers’ grains have been tested as carbon sources for the production of surfactin, but the yields were about 500 mg/L, much lower than those (> 1000 mg/L) in defined medium [20–22]. In recent years, many studies on enhancing the production of surfactin have been of particular interest. Fermentation parameters including pH, temperature, agitation speed, oxygen supply, medium composition and fermentation strategies are all important factors in surfactin production [3, 23–25]. However, with the development of genetic technology, rational engineering of strains for improved surfactin production has attracted more and more attention.

This review provides a general overview of the biosynthesis and genetic engineering strategies for enhancing surfactin production and generating novel surfactin variants. Especially, the synthetic mechanism of surfactin is not only discussed in terms of non-ribosomal peptide synthetases (NRPS); also, the biosynthesis of branched-chain fatty acids and branched-chain amino acids will be highlighted.

## Biosynthesis of surfactin and N-terminally attached fatty acids

Surfactin is one of the most prominent and well-studied members of the class of lipopeptides. Surfactin is synthesized by large multifunctional NRPS that contain three modules, SrfAA, SrfAB and SrfAC, which compose a linear array of seven modules (one module per residue) with each module being responsible for the addition of one amino acid [26, 27]. Each module contains at least three catalytic domains: an adenylation domain (A) is responsible for the selection and activation of the substrate, a small peptidyl carrier protein (PCP) carries the aminoacyl-adenylate substrate as enzyme-bound thioester, and a condensation (C) domain forms the peptide bonds between the acyl-S-PCP intermediates [28, 29]. Epimerization (E) domains perform the stereochemical conversion to yield the d-isomer of some of the incorporated residues. An additional thioesterase (TE) of the termination module catalyzes product release by either hydrolysis or macrocyclization, to generate either cyclic or cyclic-branched molecules [30, 31].

The synthesis process of surfactin can be separated into three parts, the initiation of synthesis, the elongation of the peptide chain and the cyclization of the peptide chain. The first module (C-A_Glu_-PCP) of surfactin synthetase SrfAA is responsible for the lipoinitiation reaction [32]. The donor site of the starter C domain has a very distinct specificity for the chain length of its 3-hydroxy fatty acid substrate.

Although the N-terminally attached fatty acids are key structural elements of surfactin, few researchers paid attention to their biosynthesis pathway [33–35]. Among the produced surfactin variants, those with branched-chain fatty acids are the main component, accounting for about 78% of the total [27]. Hence, the fatty acid biosynthesis system, especially that for branched-chain fatty acids, is also critical for the synthesis of surfactin in addition to NRPS. Beta-ketoacyl-acyl carrier protein synthase III (FabH) catalyzes the condensation of malonyl-acyl carrier protein (ACP) with acetyl-CoA to form β-ketobutyryl-ACP, which is the initial step of straight chain saturated fatty acid biosynthesis. However, the FabH of *B. subtilis* can initiate the straight- and branched-chain fatty acid synthesis cycle by condensing acetyl-CoA, isobutyryl-CoA, isovaleryl-CoA or α-methylbutyryl-CoA with malonyl-ACP, and it showed higher activity and selectivity for branched-chain fatty acid synthesis precursors [36, 37]. In *Bacillus*, the branched-chain fatty acid phospholipids are the main components of cell membrane phospholipids, accounting for 96% of total phospholipids (iso-C14: 0, 4%, iso-C15: 0, 24%, iso-C16: 0, 12%, iso-C17: 0, 13%, anteiso-C15: 0, 34%, anteiso-C17: 0, 9%, normal-C16: 0, 4%) [38]. The branched-chain fatty acid synthesis precursors isobutyryl-CoA, isovaleryl-CoA and α-methylbutyryl-CoA can be derived from the branched-chain amino acids l-valine, l-leucine and l-isoleucine, respectively. Thus, the biosynthesis of branched-chain amino acids and branched-chain fatty acids also greatly influences surfactin biosynthesis.

**Fig. 2:**
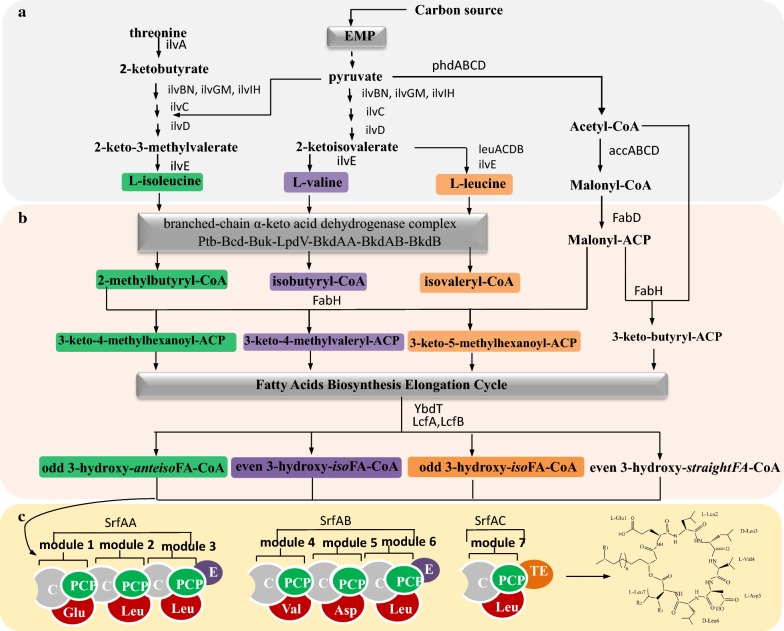
The biosynthesis pathways of branched-chain amino acids participating in surfactin biosynthesis. **a** Branched-chain amino acid biosynthesis module, represented by light grey panel. ilvA, l-threonine dehydratase; ilvBN, acetohydroxy acid synthase I; ilvGM, acetohydroxy acid synthase II; ilvIH, acetohydroxy acid synthase III; ilvC, acetohydroxy acid isomeroreductase; ilvD, dihydroxy acid dehydratase; leuACDB: leuA, 2-isopropylmate synthase; leuCD, isopropylmalate isomerase; leuB, 3-isopropylmalate dehydrogenase; EMP, Embden–Meyerhof–Parnas pathway, marked with deep gray panel; ilvE, branched chain amino acid aminotransferase; phdABCD, pyruvate dehydrogenase; accABCD, acetyl-CoA carboxylase. **b** Biosynthesis of branched-chain fatty acids and CoA-activated 3-hydroxy long chain fatty acids, represented by light orange panel. fabD, malonyl-CoA:ACP transacylase; FabH, β-ketoacyl-ACP synthases; Branched-chain α-keto acid dehydrogenase complex marked with deep gray panel; Ptb, butyryl coenzyme A transferase; Bcd, l-leucine dehydrogenase; Buk, butyrate kinase; LpdV, 2-oxoisovalerate dehydrogenase; BkdAA, 2-oxoisovalerate dehydrogenase; BkdAB, 2-oxoisovalerate dehydrogenase; BkdB, 2-oxoisovalerate dehydrogenase; YbdT, fatty acid beta-hydroxylating cytochrome P450 enzyme; LcfA and LcfB, long-chain fatty acid-CoA ligases. FAB, fatty acid biosynthesis. The degradation pathway of l-isoleucine was marked with green panels; the degradation pathway of l-valine was marked with purple panels; the degradation pathway of l-leucine was marked with orange panels. **c** Nonribosomal peptide synthetase synthesis module. A, adenylation domain, represented by amino acids in red colour; PCP, peptidyl carrier protein domains, shown in green colour; C, condensation domain, shown in gray colour; E, epimerization domain, shown in purple colour; TE, thioesterase domain, shown in orange colour

As shown in Fig. 2, the process by which the branched chain amino acids l-isoleucine, l-valine and l-leucine participate in surfactin biosynthesis was divided into three parts: branched-chain amino acids biosynthesis, branched chain fatty acid and CoA-activated 3-hydroxy fatty acids precursor biosynthesis, and NRPS-catalyzed synthesis. The biosynthesis of the branched-chain amino acids l-isoleucine, l-valine and l-leucine shares the same enzyme system encoded by ilvBN, ilvGM, ilvIH, ilvC, ilvD, and ilvE [39, 40]. Especially, the production of l-leucine from the intermediate precursor α-keto-isovalerate involves an enzyme complex encoded by leuACDB. The resulting intermediates are converted into the corresponding branched-chain acyl-CoA precursors: α-methylbutyryl-CoA, isobutyryl-CoA, and isovaleryl-CoA through the branched-chain α-keto acid dehydrogenase complex [41]. Subsequently, these branched-chain acyl-CoAs and malonyl-ACP are condensed to yield 3-keto-4-methylhexanoyl-ACP, 3-keto-4-methylvaleryl-ACP and 3-keto-5-methylhexanoyl-ACP by the action of FabH [42]. At the same time, acetyl-CoA is condensed into 3-keto-butyryl-ACP which is the precursor of straight chain fatty acids. Then, these fatty acyl precursors enter into the fatty acid biosynthesis elongation cycle to produce corresponding branched- and straight-chain fatty acids, as shown in Fig. 2 [43]. Next, the respective fatty acids are hydroxylated by the cytochrome P450 enzyme YbdT, which was proved to be responsible for the 3-hydroxylation of long chain fatty acids (LCFAs) [44, 45]. The subsequent activation of 3-hydroxy LCFAs occurs via the activity of acyl-CoA ligases LcfA and LcfB in *B. subtilis* [32]. The final CoA-activated LCFAs are recognized as substrates for the initiation of surfactin synthesis.

The importance of branched-chain amino acid and branched-chain fatty acid synthesis was recently confirmed by transcriptome analysis. The genome and transcriptome analysis of *B. amyloliquefaciens* MT45, a high-producing strain with a surfactin titer of 2.93 g/L, showed that most genes in fatty acid synthesis pathway were upregulated compared with *B. amyloliquefaciens* type strain DSM7T, which produced much less surfactin [46]. Furthermore, genes participating in acetyl-CoA generation, which is a precursor of fatty acids, were also particularly upregulated. Likewise, the results of our recent work showed that *Bacillus velezensis* BS-37 [47], a surfactin high-yield strain, were in agreement with the transcriptome analysis. Moreover, the surfactin production of strain BS-37 doubled to nearly 2 g/L with the addition of 10 mM l-Leu, which was consistent with a previous report that surfactin production could be increased 20.9-fold by strengthening the leucine metabolic pathway [35]. In addition to these findings, there were many studies that proved the importance of branched-chain amino acids and branched-chain fatty acids. For example, the deletion of the gene *lpdV*, encoding a part of the dehydrogenase complex responsible for the conversion of CoA-precursors to respective branched chain FAs, causing surfactin C14 isoform with straight FA chain was twofold more than the wild type. The research of Kraas et al. showed that the deletion of LcfA and LcfB, which were proposed to activate fatty acids for degradation, causing the production of surfactin to decrease by 84% [32].

## Strategies for enhancing surfactin production

Most reported surfactin titers of wild-type bacterial producers are in the range of 100–600 mg/L. For example, *B. subtilis* BS-37 was reported to produce 585 mg/L [16], and *B. velezensis* H3 488 mg/L [48]. It has been difficult to achieve significant breakthroughs in production only through traditional strategies of mutagenesis and breeding or fermentation optimization. Consequently, establishing genetically modified surfactin producer strains is of great significance. In terms of strain modification to improve the production of surfactin, there are mainly three strategies: (1) substituting the native promoters P_srf_ of *srfA* modules, which is important for surfactin synthesis; (2) strengthening the efflux of surfactin by overexpression of assistant proteins and surfactin transporters is also an effective way. (3) To modify the transcriptional regulatory genes of *srfA.*

### Promoter engineering

The production of surfactin requires the critical *srfAB* locus, which is a large operon of 27 kb controlled by the promoter P_srf_. Because it is difficult to heterologously express *srfA*, promoter exchange has been regarded as a preferred way to improve the productivity of surfactin. There are three types of promoters often used in *B. subtilis*: inducer-specific promoters, constitutive promoters and autoinducible promoters [49]. The best-known constitutive promoter is P_43_. The inducible promoter P_xyl_ is driven by xylose, P_spac_ is induced by IPTG. Auto-inducible promoters (e.g., P_pst_ and P_cry3Aa_) can be used to express the target gene from the late log phase to the stationary phase [50]. P_srf_ is an autoinducible promoter which is triggered by signal molecules acting in a quorum sensing pathway. The recent successes in promoter exchange to promote the yield of surfactin were achieved using natural high-yield strains [51, 52]. In an earlier study, Sun et al. used the IPTG-inducible hybrid promoter P_spac_ to replace the native P_srf_ promoter of *B. subtilis* fmbR, which resulted in a tenfold surfactin yield enhancement, to about 3.86 g/L [53]. In addition to using natural strong promoters, synthetic promoters were also investigated, and perhaps the most remarkable result was achieved by Song et al. [51]. They identified several strong native promoters (P_groE_, P_sacB_ and P_sacP_) in *B. subtilis* THY-7 through transcriptome analysis and confirmed the weakness of the native *srfA* promoter. However, the recombinant strains could not successfully synthesize surfactin using the strong constitutive P_groE_ core promoter. Afterwards, three novel promoters were designed using the P_groE_ core promoter as the basis. When the ultra-strong chimeric promoter P_g3_ was used to drive surfactin synthesis, the surfactin titer in flasks reached 8.61 g/L, which was 15.6-fold greater than that of wild-type THY-7.

However, there are also reports of failures of promoter modification in model strains or other organisms. Coutte et al. reported that the surfactin concentration changed from 1.5 to 1.2 g/L after replacing the P_srfA_ of the *B. subtilis* 168-derived strain BBG111 with the constitutive promoter P_repU_ originating from the replication gene *repU* of the *Staphylococcus aureus* plasmid pUB110 [54]. Subsequently, Willenbacher et al. analyzed the effect of promoter replacement in minor and strong surfactin producer strains. They found the surfactin concentration of minor producer strains *B. subtilis* 3A38 was increased, while the strong producer strains *B. subtilis* DSM 10T was decreased after substitution of the native promoter using constitutive promoter P_veg_, as shown in Table 1 [52].

**Table 1:** Surfactin yields of recombinant strains

Strain	Description	Production (g/L)	References
*B. subtilis* fmbR	Native P_srfA_	0.38	[53]
*B. subtilis* fmbR-1	Replacement of P_srfA_ with P_spac_	3.86	
*B. subtilis* THY-7	Native P_srfA_	0.55	[51]
*B. subtilis* THY-7/Pg3-*srfA*	Replacement of P_srfA_ with P_g3_	9.74	
*B. subtilis* BBG111	Native P_srfA_	1.5	[54]
B. subtilis BBG113	Replacement of P_srfA_ with P_repU_	1.2	
*B. subtilis* 3A3B	Native P_srfA_	0.07	[52]
*B. subtilis* JWSurf2	Replacement of P_srfA_ with P_veg_	0.26	
*B. subtilis* DSM 10T	Native P_srfA_	0.62	[52]
*B. subtilis* JWSurf3	Replacement of P_srfA_ with P_veg_	0.04	
*B. subtilis* THY-7	–	0.55	[57]
*B. subtilis* TS589	Overexpression of THY-7-P_grac_-*ycxA*	1.15	
*B. subtilis* TS593	Overexpression of THY-7-P_grac_-*krsE*	0.93	
*B. subtilis* TS662	Overexpression of THY-7-P_grac_-y*erP*	1.67	
*B. subtilis*	–	0.021	[69]
*B. subtilis* (pHT43-*comXphrC*)	Overexpression of ComX and PhrC	0.135	
*B. subtilis* BBG258	Insertion of the *sfp* gene in the *amyE* locus of *B. subtilis* 168	0.221	[35]
*B. subtilis* BBG260	Knocking out *codY* in BBG258	2.289	

Overall, to establish genetically modified high-yield surfactin producer strains by promoter engineering, it is necessary to analyze the transcriptome and production capacity of the strain. Targeted construction of a strong promoter or hybrid promoter suitable for each strain may be more suitable. However, it is also necessary to consider that there are many uncertainties in the genetic operating system of wild-type strains, which greatly increases the difficulty of genetic manipulation.

### Enhancement of the efflux of surfactin

The mechanism guiding the transmembrane efflux of surfactin is not very clear, but surfactin has a deep effect on the lipids of biological membranes [55]. As reported, the surfactin monomer can insert itself into phospholipid layers in biomimetic membrane systems at low concentrations (below or near the critical micelle concentration, CMC). At higher concentrations, surfactin can cause membrane solubilization and vesicle destruction [56]. Some researchers speculated that the transmembrane efflux of surfactin may simply be the result of membrane insertion and penetration of surfactin monomers or oligomers [57]. In an early report, Tsuge et al. [58] suggested that there may be a mechanism for surfactin efflux mediated by protein transporters. They found that the production and resistance to surfactin of *B. subtilis* hosts could be significantly reduced by null mutations in *yerp*, a gene encoding a protein with homology to RND (resistance, nodulation and cell division) family efflux pumps. The speculation that transmembrane exporters dependent on the proton motive force (PMF) could facilitate the efflux of surfactin in *B. subtilis* was further confirmed by Li et al. According to the energy source, bacterial transmembrane transporters can be divided into two categories, those dependent on ATP or proton motive force (PMF, i.e. transmembrane proton concentration gradient) [59]. Firstly, Li et al. used liposomes and transmembrane transport inhibitors to confirm that the surfactin efflux in THY-7 was mainly dependent on the PMF, and not ATP hydrolysis. Then, they identified the putative lipopeptide transporter YcxA, which depends on the PMF, unable to transfer surfactin in THY-7 due to a frameshift mutation of the encoding gene. Afterwards, three putative lipopeptide transporters with PMF as energy source were overexpressed, and the secretion of surfactin increased by 89%, 52% and 145% with the overexpression of the natural full-length YcxA, KrsE and YerP proteins.

To enhance the efflux of surfactin, proteomics can be used to analyze the differences between key proteins related to efflux in different host strains, especially wild-type strains with high productivity of surfactin. However, the ability of surfactin efflux in the strain needs to be identified first. The mechanism of surfactin efflux and proteins involved in transmembrane transport still need to be further researched.

### Modifying the transcriptional regulatory genes of *srfA* operon

**Fig. 3:**
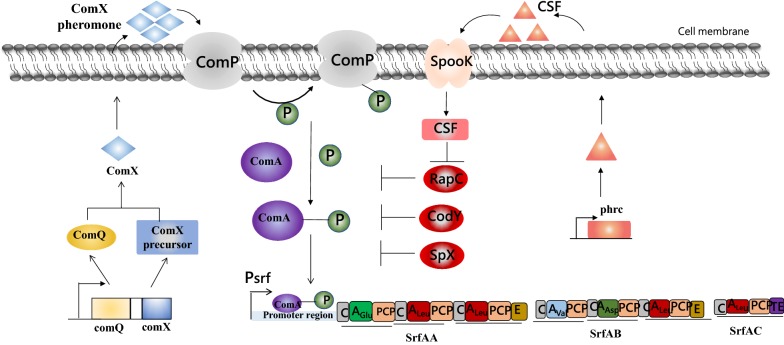
The schematic model for the regulation of the transcription of the *srfA* operon network involved in two extracellular signaling peptide-mediated quorum sensing in *B. subtilis*. T-bars indicate the negative effects on DNA binding or protein interactions. Bent arrow represents the promoter. ‘P’ in the circle represents the phosphoryl group

The expression of *srfA* operon is not only controlled by the promotor P_srf_, but also can be regulated by some transcriptional regulatory genes. Bacterial quorum sensing (QS) system, a cell-density-dependent regulatory networks, plays a significant role in the regulation of *srf*A operon. The two canonical peptides, ComX and CSF, mediate the quorum sensing control of competence and sporulation in *B. subtilis* [60, 61]. In the late-growth phase, a series of gene transcription events downstream of the ComA-ComP two-component are triggered by the accumulation of a high concentration of peptides, among which the *srfA* operon is stimulated via a complex-regulation phosphorylated cascade [62–64]. As shown in Fig. 3, both ComX and CSF activate the transcription of the *srfA* operon by stimulating the activity of transcription factor ComA through phosphorylation (ComA-P) via two separate pathways. In one pathway, the monitor of cell density, the ComX pheromone, is firstly modified by the ComQ isoprenyl transferase to be processed outside. The signal transduction system composed of the two-component regulatory proteins ComP and ComA is activated when the ComX pheromone reaches the obligatory concentration. Ultimately, phosphorylated ComA binds to the promoter (P_srfA_) of the *srfA* operon in tetramer form, and cooperates with SigA to activate the transcription of corresponding gene expression [65]. Another pathway is mediated by CSF, which is encoded by *phrC* and is initially imported as inactive-form CSF (inact). CSF is imported into the cell by the oligopeptide permease SpooK [66], and then binds to the Rap protein, which causes the Rap protein losing phosphatase activity. Thereby, the dephosphorylation of phosphorylated ComA can be prevented to facilitate the transcription of *srfA* gene and synthesis of surfactin [67].

In early research, the expression of *srfA* was decreased by inhibiting the transcription of the ComQXP quorum-sensing locus in *B. subtili*s [68]. In addition, Wang et al. found that surfactin production was severely decreased by a mutation of three non-aspartate amino acids in the ComA response regulator receiver motif. Later, Jung et al. confirmed that the overexpression of signaling factor ComX and PhrC could successfully increase the production of surfactin [69].

In addition to the ComX and CSF, the expression of the *srfA* operon is also regulated through several global regulators and regulatory protein. The global regulator CodY can repress *srfA* transcription through directly binding to the *srfA* promoter regions [70, 71]. The *codY*-dependent repression of *srfA* transcription can be triggered by high external concentrations of amino acids. Consequently, the surfactin production in *B. subtilis* 168 derived strains was increased about tenfold through the knockout of *codY* [35]. The regulatory protein SpX suppresses *srfA* expression by blocking the interaction between ComA and RNA polymerase (RNAP) in the promoter region via competition for an overlapping site in the α-CTD [72]. Some other negative regulators of *srfA* was also detailly reported such as PerR, SinI and PhoP [33, 46, 73, 74].

The synthesis of surfactin is closely related to QS, and ComX seems to be a key factor in enhanced surfactin productivity. However, not much is known about the dynamics of the regulatory network or overall synthesized quantities. At present, the surfactin productivity of strains used in studies on the synthesis of QS regulatory genes is generally low. Thus, whether these QS regulatory genes and their regulation in high-yield strains have changed, as well as if there exist other signal peptides like PhrC, awaits further study.

### The genomics and transcriptomics analysis assisted rational strain improvement

**Fig. 4:**
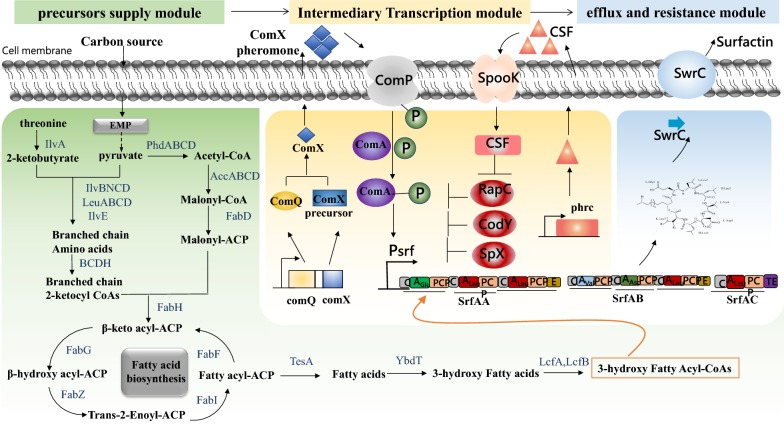
The general regulatory network of surfactin synthesis. Refer to Wu et al. [33], with minor modifications

Genome sequencing in combination with global transcriptome analysis is an effective strategy to unravel the biosynthesis and regulatory features of surfactin exhibited in high-producing strains; They can provide fundamental information for rational strain improvement via genetic modification or pathway engineering. The general regulatory network of surfactin synthesis could be divided into three functional modules: precursors supply module, intermediary transcriptional driving module and efflux and resistance module, as shown in Fig. 4.

Compared to low-producing strain *Bacillus amyloliquefaciens* DSM7^T^ [75], the up-regulated expressed genes in high-producing strain *B. amyloliquefaciens* MT45 mainly be involved in srfA operon expression, precursor redirection, and antibiotic resistance capacity. For example, the *srfA* operon was up-regulated about 9.25–48.86-fold in high-producing strain *B. amyloliquefaciens* MT45 [46]. Apart from this, most genes in the fatty acid synthesis pathway, such as *fabG*, *fa*bZ, *fabI* and *fabF* were also up-regulated. Moreover, in efflux and resistance module, *swrC* (synonymous of *yerP*) and other two genes *acrB* and *liaRSFGHI* operon were all highly expressed. AcrB had high amino acid sequence similarity to SwrC (~ 41%) and *liaRSFGHI* operon was annotated as genes associated with the resistance of daptomycin (a structural analogue of surfactin). Meanwhile, the *srfA* operon and *swrC* were also highly expressed in high-yield strain *Bacillus velezensis* BS‐37 [47].

Most recently, Wu et al. [33] used *B. subtilis* 168 as the initial host to construct a surfactin hyperproducer through a systematic metabolic engineering method based on the results of genomics and transcriptomics of *Bacillus amyloliquefaciens* MT45. Three functional modules in *B. subtilis* 168 were strengthened via more than 40 steps of manipulations; the final surfactin titer reached up to 12.8 g/L. The surfactin production of this functional strain is also the highest yield reported so far. Compared with the wild strain *B. subtilis* THY-7 derived high producer recombinants (9.74 g/L), we can speculate that the wild high producers could be more efficient for constructing a surfactin hyperproducer for industrial application, if efficient genetic manipulations were available.

## Structural modification of surfactins

In addition to the use of genetically engineered surfactin-producing strains to enhance the production, some strategies were developed to modify the structures of surfactins. Combinatorial biosynthesis, a genetical engineering technique that purposefully alters the biosynthetic pathway of natural products to form predictable new structural products, plays an important role in the structural modification of cyclolipopeptide antibiotics [76]. These novel structural compounds exhibit new functions or activities as expected by the investigator. The strategies for structural modifications are mainly focused on the peptide ring and hydrophobic fatty acid chain.

### Surfactin variants with novel peptides

The peptide ring of seven amino acids in surfactin is synthesized by NRPS modules. The site-directed mutagenesis, substitution, insertion, deletion, and reprogramming of peptide modules within a NRPS biosynthetic template can provide an almost infinite recombination potential to generate novel peptides. Under these circumstances, various approaches have been tested to rationally design novel surfactin products, as shown in Table 2. Some modifications were mainly focused on the substrate specificity of NRPS. Previously, the modification of surfactin was conducted by rational module swapping, such as the targeted replacement of the simplest peptide synthetase module SrfA-C [77, 78]. Later, Stachelhaus et al. extended this method to exchange the leucine-activating module within the multi-modular SrfA-A [79]. As a result, ornithine was directly incorporated at the second position of the peptide chain. However, the substitution caused a more substantial modification in the conformation of the peptide product. For example, through structural dissection of the phenylalanine-activating adenylation (A) domain, which is responsible for the specific recognition of the cognate substrate amino acid in nonribosomal peptide synthetases (NRPSs), Eppelmann et al. rationally altered the substrate selectivity of the initiation module C-A_Glu_-PCP of the surfactin synthetase complex from l-Glu to l-Gln using site-directed mutagenesis [80]. What’s more, a novel surfactin with an l-Asn residue at position 5 replacing the native l-Asp constituent was produced in the same way. Moreover, the NRPS was reprogrammed to be more “clickable” by a single tryptophan-to-serine mutation in the phenylalanine-specific NRPS adenylation domain, which enabled the efficient activation of non-natural aromatic amino acids functionalized with azide and alkyne groups [81]. These results greatly enriched the diversity of peptide modules by “click” reactions, which represent Huisgen cyclizations between alkynes and azides.

**Table 2:** Novel surfactin variants with modifications of the peptide ring

Strain	Modification	Variants	Yields (mg/L)	Properties	Reference
*B. subtilis* ATCC2133	Substitution	[Cys^7^]	–	Decreased hemolytic activity	[77]
*B. subtilis* ATCC21332	Substitution and deletion	1. [Δ(Val^4^-Leu^3^)Orn^2^] 2. [Δ(Val^4^)Orn^2^] 3. [Δ(Leu^7^-Leu^6^)Orn^2^] 4. [Δ(Leu^7^)Orn^2^]	10–20% of original strain	Decreased hemolytic activity	[79]
*B. subtilis* KE100	Mutagenesis and substitution	1. [Gln^1^] 2. [Asn^5^]	–	–	[80]
*B. subtilis* ATCC	Deletion	[ΔLeu^2^]	25–50	Higher antibacterial ability and decreased hemolytic activity	[82]
*B. subtilis* BP2-L1	Deletion	1. [ΔLeu^3^] 2. [ΔLeu^6^] 3. [ΔAsp^5^]	0.82–1.35	[ΔLeu3] and [ΔLeu6] with antifungal activity and reduced toxicity; [ΔAsp5] with higher antimicrobial activity	[83]
*B. subtilis* OKB10	Deletion	[FA-Glu]	200–250	Lower CMC and higher water solubility	[84, 85]

“–” means not mentioned

Decreasing the ring size by means of genetic engineering is another way to produce novel peptide products. Researchers obtained a condensed ring product by deleting the leucine-incorporating SrfA-A2 module of the surfactin NRPS, resulting in a hexapeptide, Δ2-surfactin variant with a decreased ring size [82]. The hexapeptide had reduced toxicity to erythrocytes with higher antibacterial ability. However, due to the instability of the structure, the yield of hexapeptide surfactin was only 5–10% that of the wild-type product, about 25–50 mg/L. Likewise, Jiang et al. knocked out the modules SrfA-A-Leu, SrfA-B-Asp, and SrfA-B-Leu of the surfactin NRPS in *B. subtilis* BP2-L1, respectively [83]. These modifications resulted in three novel hexapeptide surfactins, individually lacking the amino acids Leu-3, Asp-5, and Leu-6. Although [ΔLeu^3^]surfactin and [ΔLeu^6^]surfactin showed reduced toxicity, and the [ΔAsp^5^]surfactin showed stronger antimicrobial activity than the native surfactin, the yields of the novel products were extremely low, only 0.82–1.35 mg/L. Moreover, the size of the peptide ring could be minimized by the deletion of six amino acids, with only a single glutamic acid residue remaining. This surfactin variant, fatty-acyl-glutamate (FA-Glu) had a lower CMC and higher water solubility than myristoyl glutamate, a commercial surfactant. However, the yield of FA-Glu was also low, reaching only about 200–250 mg/L which was 5% of that of the wild-type product [84, 85].

Combinatorial biosynthesis technology presents both opportunities and challenges in developing new surfactant structures and expanding their applications. The reprogramming and deleting peptide modules of surfactin can result in many novel surfactin products with new or better capabilities, but many of the reported engineering attempts faced low product yields or even inactive hybrid enzymes. Indeed, the complexity of the NRPS structure far exceeds people’s imagination. The integrity of the modules and the diversification of the link area always brings uncertainty to the genetic manipulation. The extensive unexpected modification of the peptide-product and the low yield all need to be overcome.

### Modification of the fatty acyl structure of surfactin

Compared with the NRPS-catalyzed synthesis of the peptide structure, the biosynthesis of the fatty acyl structure of the surfactin molecule still needs further research. The fatty acid chain length of surfactin is generally C_13_–C_15_, whereby C_14_ and C_15_ are usually predominant (60–80%) [34]. The length and isomerism of the FA chain were known to have an impact on the physicochemical properties and biological activity [86].

The antifungal activity and surface activity was enhanced in the order straight (n) > iso > anteiso [86, 87]. The C_14_ surfactin was found to have higher foaming capacity in comparison to C_13_ and C_15_ surfactins [45, 88]. It was also found that a linearized synthetic C_14_ surfactin showed none or less hemolytic behavior in comparison to cyclic natural C_14_ surfactin [89]. The fatty acid composition of surfactin has a greater effect on the surface activity than on the surfactin production. Iso-odd fatty acid isomers have higher oil displacement than n-even fatty acid isomers [27]. Our research also proved if the surfactin contained more proportion of C_15_, the surfactin showed a better oil-washing efficiency and oil displacement efficiency [16]. Therefore, in the study of the structural diversity of surfactin, the directional modification of the fatty acid chain of the surfactin product is of great significance for the application of surfactin.

As illustrated in Fig. 2, branched-chain amino acids are closely related to the fatty acyl moiety of surfactin. Hence, amino acids added to the culture medium affect not only the amino acid moiety in the peptide ring but also the hydroxyl fatty acid moiety in the produced lipopeptide. For example, adding Arg, Gln or Val to the medium increased the proportion of surfactin with even β-hydroxy fatty acid components C_14_ and C_16_, whereas the addition of Cys, His, Ile, Leu, Met, and Ser enhanced the odd β-hydroxy fatty acids in *B. subtilis* TD7 [90]. Since then, people began to change the fatty acid composition of the surfactin products by modifying or strengthening the relevant amino acid synthesis pathways. Coutte et al. increased the production of surfactin via intracellular l-leucine overproduction by genetically engineering *B. subtilis* 168 [35]. Notably, not only the yield of surfactin was maximized, but the structural components of surfactin were also changed after knocking out the global regulatory factor CodY, which inhibits the expression of the *ilv*-*leu* operon. The relative proportions of C_13_ and C_14_ in the control strain were 39.7% and 21.2%, respectively, which changed to 26.5% and 40.6% after knocking out *codY*. Later, Coutte et al. further studied the branched-chain amino acid metabolic pathway and found that the proportion of surfactin with linear C_14_ was increased 2.5 times after knocking out the *lpdV* gene responsible for the final degradation of branched-chain amino acids [34].

Obviously, it is difficult to produce directional fatty acid structures of surfactin by using exogenously added amino acid components or internally engineered amino acid synthesis pathways. In order to purposefully modify the structure of fatty acids, it is necessary to understand how the 3-hydroxy fatty acids participate in the lipoinitiation reaction of surfactin biosynthesis. Kraas et al. revealed the mechanism of lipoinitiation by functional dissection of surfactin synthetase [32]. In the initial step of surfactin biosynthesis, the substrate 3-hydroxy fatty acid is combined with coenzyme A (CoA) under the control of fatty acyl CoA ligases (FACLs), which activates the acylation of the first amino acid Glu in the form of a CoA-activated 3-hydroxy fatty acid. Furthermore, gene deletion studies of four putative fatty acyl CoA ligases (FACLs) showed that the yield of surfactin was reduced by 38–55% with a single knockout of one gene, while the surfactin yield was about 20–65% when 2–3 genes were knocked out, and decrease by 84% when the four putative fatty acyl CoA ligases were all knocked out. These results showed that fatty acyl CoA ligases have a significant effect on surfactin biosynthesis. It should also be noted that deactivating the four putative FACLs does not result in a complete loss of the ability to synthesize surfactin. It therefore seems that there are still fundamental discoveries to be made, more FACLs need to be found and there are still other pathways providing the fatty acid moiety for surfactin production, for example, through transthiolation from ACPs to CoA.

The pool of fatty acid-S-CoA substrates contains many different CoA-activated 3-hydroxy fatty acids which are not all incorporated into surfactin. The SrfAA starter C-domain can catalyze the direct formation of an amide bond between a fatty acyl-CoA and an amino acid moiety. Thus, the substrate selectivity of the C-domain in the initiation module (C-AGlu-PCP) of surfactin synthetase SrfAA plays a more important role in the fatty acyl structure of surfactin. This will give us new insights for the rational design and genetic modification of the fatty acyl structure of surfactin. The C domain of the NRPS synthesis initiation module srfA-A1 may be an important target for fatty acyl structural modification. Changing the specificity of the starter C-domain for fatty acids or swapping the starter C-domains in the initiation module of surfactin synthetase SrfAA for those from foreign NRPSs is a potential way to generate novel surfactin structures with a specific fatty acyl moiety. The exciting work by Kraas et al. was confirmed by Chooi and Tang, who demonstrated that the starter C-domain of SrfAA synthetase is really a worthwhile research subject [91]. Regrettably, there have been no further reports investigating C-domain modification.

## Conclusion and prospects

Surfactin has attracted considerable attention in research and industrial applications due to its various biological and physico-chemical properties, but the current fermentation processes are cost-prohibitive and cannot meet the needs of industrial applications. Controlling the foaming during the fermentation process remains a challenge in the industrial fermentation of biosurfactants. Loss of product, nutrients, and cells caused by foam overflow needs to be solved through process- and reactor optimization. In addition, finding cheap biomass materials is also an effective means to reduce the cost of fermentation and promote the industrial application of surfactin.

With advances in genetic engineering and synthetic biology, the construction of strains with high yield, high conversion rate and high production rate will become feasible in the near future. Creating novel surfactin compounds with new structures is also the key to promote their broader application. The studies on NRPS modules and combinatorial biosynthesis technology provide the basis for the modification of the surfactin peptide loop. However, there are still many hurdles to overcome, including the functional connection between modules, uncertainty about the effects of the modified peptide products, and the low yields. On the other hand, there are still no technical means that would enable the directed regulation of the fatty acyl structure of surfactin. Enhancing the de-novo synthesis pathway of fatty acid precursors may be a good strategy to increasing the production of surfactin with a specific fatty acyl structure. The substrate specificity of the initiation module (C-AGlu-PCP) needs to be analyzed further.

## Abbreviations

CLPs: cyclic lipopeptides
EOR: enhanced oil recovery
MEOR: microbial enhancement of oil recovery
FHW: feather hydrolysate waste
GMW: glutamate mill waste
NRPS: nonribosomal peptide synthetase
LCFAs: long chain fatty acids
PMF: proton motive force
QS: quorum-sensing
FACLs: fatty acyl CoA ligases
FA-Glu: fatty-acyl-glutamate
CoA: coenzyme A
